# In-line filtration reduces severe complications and length of stay on pediatric intensive care unit: a prospective, randomized, controlled trial

**DOI:** 10.1007/s00134-012-2539-7

**Published:** 2012-04-12

**Authors:** Thomas Jack, Martin Boehne, Bernadette E. Brent, Ludwig Hoy, Harald Köditz, Armin Wessel, Michael Sasse

**Affiliations:** 1 Department of Pediatric Cardiology and Intensive Care Medicine, Hannover Medical School, Carl-Neuberg-Strasse 1, 30625 Hannover, Germany; 2 Department of Pediatrics, Faculty of Medicine, Imperial College, London, UK; 3 Institute of Biometrics, Hannover Medical School, Hannover, Germany

**Keywords:** In-line filtration, SIRS, Intensive care, Particle, Inflammation, Children, Complication

## Abstract

**Purpose:**

Particulate contamination due to infusion therapy carries a potential health risk for intensive care patients.

**Methods:**

This single-centre, prospective, randomized controlled trial assessed the effects of filtration of intravenous fluids on the reduction of complications in critically ill children admitted to a pediatric intensive care unit (PICU). A total of 807 subjects were randomly assigned to either a control (*n* = 406) or filter group (*n* = 401), with the latter receiving in-line filtration. The primary endpoint was reduction in the rate of overall complications, which included the occurrence of systemic inflammatory response syndrome (SIRS), sepsis, organ failure (circulation, lung, liver, kidney) and thrombosis. Secondary objectives were a reduction in the length of stay on the PICU and overall hospital stay. Duration of mechanical ventilation and mortality were also analyzed.

**Findings:**

Analysis demonstrated a significant reduction in the overall complication rate (*n* = 166 [40.9 %] vs. *n* = 124 [30.9 %]; *P* = 0.003) for the filter group. In particular, the incidence of SIRS was significantly lower (*n* = 123 [30.3 %] vs. *n* = 90 [22.4 %]; *P* = 0.01). Moreover the length of stay on PICU (3.89 [95 % confidence interval 2.97−4.82] vs. 2.98 [2.33−3.64]; *P* = 0.025) and duration of mechanical ventilation (14.0 [5.6−22.4] vs. 11.0 [7.1−14.9] h; *P* = 0.028) were significantly reduced.

**Conclusion:**

In-line filtration is able to avert severe complications in critically ill patients. The overall complication rate during the PICU stay among the filter group was significantly reduced. In-line filtration was effective in reducing the occurrence of SIRS. We therefore conclude that in-line filtration improves the safety of intensive care therapy and represents a preventive strategy that results in a significant reduction of the length of stay in the PICU and duration of mechanical ventilation (ClinicalTrials.gov number: NCT00209768).

**Electronic supplementary material:**

The online version of this article (doi:10.1007/s00134-012-2539-7) contains supplementary material, which is available to authorized users.

## Introduction

The intravenous administration of fluids and drugs is essential in the management of critically ill patients. The contamination of infusion solutions by particles is a widely unknown and underestimated side effect of intravenous therapy [1, 2]. Particulate contamination is due to drug incompatibility reactions or their incomplete reconstitution during the preparation process [3]. Various studies have demonstrated the contamination of infusion solutions with glass particles from opening glass ampoules, particles from rubber stoppers or conglomerates of the parenteral nutrition components [4, 5]. Particles have also been shown to be inherent to generic drug formulation [2]. In an intensive care setting the particle burden may rise up to one million infused particles per day, increasing with the complexity and quantity of the administered infusions [6, 7]. Acknowledging the risks associated with such contamination, it is important to optimize infusion therapy in order to minimize medication errors and particle load. Therefore, standard operational procedures for the infusion set-up, databases for the prevention of drug incompatibilities and training of medical staff can be helpful tools to improve patient safety [3, 8, 9]. In addition, in-line filtration has been shown to almost completely prevent particulate infusion [4]. Two intravenous fluid filters are currently in widespread use: 0.2-μm filters for crystalline solutions and 1.2-μm filters for lipid-containing admixtures. Positively charged 0.2-μm filters are able to retain particles, air and micro-organisms and endotoxins [10].

Post-mortem examinations of adults suffering from acute respiratory distress syndrome (ARDS) [7] and children with underlying disease [11] have revealed that infusion therapy can lead to particle-induced mechanical blockage of vessels and the generation of pulmonary foreign body granulomata. Particles harm the pulmonary endothelium either directly or through activation of complement, platelets and/or neutrophils, leading to the formation of occlusive microthrombi [7, 12]. In vitro studies with endothelial cells and macrophages have also demonstrated particle-induced modulation of immune response [5]. The effect of particle infusions is aggravated in situations of disturbed microcirculation, such as ischemia and reperfusion injury: after cardioplegia, particles in cardioplegia solutions lead to impaired coronary artery flow, alterations in endothelium and increased leukocyte adhesion in humans as well as in animals [1]. In an experimental rat liver transplantation model, particles in the preservation solution were found to disturb the microcirculation and aggravate post-ischemic inflammation [13]. In a hamster skinfold chamber model, following ischemia, intravenous infusion of particle-contaminated solutions reduced functional capillary density by almost 50 %, compared to post-ischemic values before particle injection [14, 15]. However, filtration of solutions through 0.2-μm in-line filters prevented the loss of capillaries completely [15] and attenuated the inflammatory process [1, 13].

Previous clinical trials of in-line filtration primarily focused on the retention of micro-organisms, demonstrating a preventive effect on thrombophlebitis [16] without any impact on central venous catheter-related sepsis [17]. To date, no clinical trial has taken account of the results of experimental studies on particle contamination. However, a clinical effect of particle retentive in-line filtration would be expected to be most evident in situations of altered microcirculatory homoeostasis or immune response. Intensive care patients who experienced trauma, major surgery or systemic inflammation suffer from conditions that affect the microcirculation of tissues and vital organs [18].

This single-centre, prospective, randomized, controlled trial in critically ill children was conducted to evaluate the impact of in-line filtration on severe complications, such as systemic inflammatory response syndrome (SIRS), sepsis, thrombosis and different organ failure.

## Materials and methods

### Study design

This was a single-centre, prospective, randomized, controlled trial conducted between February 2005 and September 2008 in an interdisciplinary pediatric intensive care unit (PICU) of a tertiary German hospital. Approval of the study protocol was obtained from the local ethics committee. Funding was primarily provided by a research grant of Hannover Medical School and partially by an unrestricted grant from Pall, Dreieich, Germany and B. Braun Melsungen, Germany. Both companies supplied in-line filters. An open label study design characterized by visible in-line filters was necessary to ensure maximum safety in drug administration and to allow nurses to monitor the in-line filters for imminent blockage. Any adverse events relating to in-line filtration were recorded.

### Patient enrolment and randomization

**Fig. 1:**
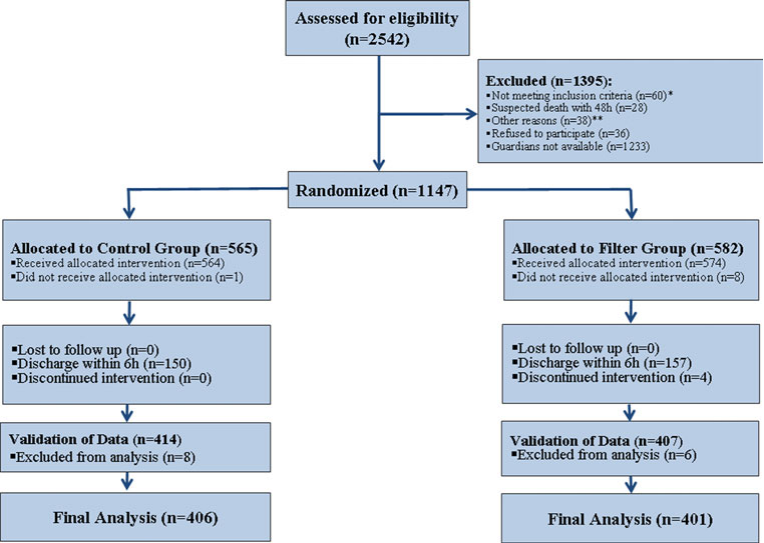
Enrolment of study subjects.* Single asterisk* denotes exclusion criteria: recruitment for other studies, 18 years of age or older, no infusion therapy during the stay in the pediatric intensive care unit (PICU).* Double asterisk* denotes other reasons for exclusion: no informed consent due to foreign language and ethical reasons. In four patients allocated to the filter group the intervention was discontinued. One patient in the control group and 8 patients in the filter group were excluded due to incorrect allocation. Fourteen patients (8 control, 6 filter group) were excluded during the final validation because of incomplete data in the medical charts

All patients younger than 18 years of age who were admitted to the PICU during the study period were eligible for enrollment (*n* = 2,542). Subjects expected to die within 48 h of admission, such as those under cardiopulmonary resuscitation, and patients already recruited for other trials or without any intravenous therapy were excluded (Fig. 1). Written informed consent was obtained for each child from their legal guardians on admission. A total of 1,147 patients met the inclusion criteria. These patients were randomly allocated to either the control or filter group based on a computer-generated simple unrestricted randomization list. Discharge within 6 h after admission was defined as an exclusion criterion. Fourteen patients (8 control, 6 filter group) were excluded during final validation due to missing relevant clinical data in their charts. The final analysis included 807 patients (343 female, 464 male) (Fig. 1).

### Infusion management and in-line filtration

Prior to this study, the infusion regimen was standardized for patients in both groups. A computer database containing information on compatibilities of drugs (KiK 3.0; ODATA, Rastede, Germany) was used to prevent incompatibilities [3]. Medications were prepared according to the manufacturer’s instructions. Parenteral nutrition and certain drugs (special antibiotics, antiviral drugs, antimycotics, chemotherapy) were supplied by a centralized intravenous additive service (CIVA) to guarantee chemical stability and aseptic standards. The filter group received in-line filtration throughout the period of infusion therapy, and all fluids except blood, plasma proteins or fresh frozen plasma were administered via in-line filters. The appropriate filters (1.2-μm pore size [Intrapur Lipid/Intrapur Neonat Lipid; B. Braun Corporation, Melsungen, Germany] for infusion of lipid-containing admixtures; 0.2-μm pore size positively charged filters [ELD96LLCE/NEO96E; Pall, Dreieich, Germany] for aqueous solutions) were arranged in the lumen of each central and peripheral venous catheter (Fig. 1 of the Electronic Supplementary Material [ESM]). Nurses checked the in-line filters at least every hour for imminent blockage. Filters were replaced after 24 h (Intrapur Lipid/Intrapur Neonat Lipid) or 72 h (ELD96LLCE/NEO96E) of regular use or in case of blockage. In both groups, the administration sets for lipid-containing admixtures were changed every 24 h, others every 72 h as recommended by the Robert Koch Institute [19]. Nurses and physicians were extensively trained prior to the study to ensure correct and safe in-line filter handling and a competent infusion management.

### Endpoints

Given the low mortality in PICU, we chose reduction in the overall complication rate of major events as the primary endpoint. Major events included the incidence of SIRS, sepsis, thrombosis, acute liver failure, ARDS and acute renal and circulatory failure. The occurrence of at least one of these major events during the PICU stay accounted for one complication per patient in the calculation of the overall complication rate. Complications prior to PICU stay and on admission were not taken into account. Sepsis and SIRS were defined according to International Pediatric Sepsis Consensus Conference (IPSCC) 2005 [20, 21]. All other endpoints were defined according to accepted pediatric consensus recommendations [22–25]. Further information is provided in the ESM. Secondary endpoints were reduction of length of stay on PICU and overall hospital stay.

### Data collection and statistical analysis

Detailed information on data collection is provided in the ESM. Statistical analysis was performed on an intention-to-treat basis. In a preceding survey a complication rate of approximately 40 % was determined. The study was designed using a chi-square test for equal proportions and with 80 % power to detect a reduction from 40 to 30 % in the complication rate for the filter group. At a significance level of 0.05, based on the previously determined complication rate, this design required 376 subjects to be enrolled in each group. The study design included an interim analysis after recruitment of 50 % of the targeted enrolment to evaluate any possible adverse effects of in-line filtration. Statistical analysis was performed with the use of Predictive Analysis Software for Windows, ver. 18 (SPSS, Chicago, IL). Further information on the statistics is provided in the ESM.

## Results

### Subjects

The scheduled interim analysis identified no termination criteria after inclusion of 50 % of the patients eligible for enrolment, and ethical approval was obtained to continue recruitment. Ultimately, 807 children were randomly assigned to either the control group (*n* = 406) or filter group (*n* = 401) and included in the final analysis. Both groups were well matched, with a similar distribution of baseline demographic characteristics and underlying disease categories, and showed no differences in the Pediatric Index of Mortality II (PIM II) calculations (Table 1).

**Table 1:** Baseline characteristics of patients

Characteristics	Control group (*n* = 406)	Filter group (*n* = 401)	*P* value^a^
Age (years)	5.58 ± 5.59	6.07 ± 6.01	0.23
Weight (kg)	21.8 ± 20.1	23.0 ± 20.7	0.43
Pediatric Index of Mortality II (PIM II)	4.15 ± 8.76	3.42 ± 9.14	0.25
Sex (*n*)			
Male	230	234	0.72
Female	175	168	
Disease category on admission (*n*)			
Cardiology	150	155	0.66
Cardiac bypass	101	102	0.87
Non-bypass	49	53	0.67
Hematology/oncology	24	21	0.76
Elective	13	11	0.84
Non-elective	11	10	1.00
Nephrology	18	26	0.21
Renal transplantation	13	19	0.28
Gastroenterology	37	37	1.00
Liver transplantation	20	19	1.00
Pulmonology	21	18	0.74
Elective	13	11	0.84
Non-elective	8	7	1.00
Pediatric surgery	59	48	0.30
Elective	56	44	0.24
Non-elective	3	4	0.72
Traumatology	34	43	0.28
Elective	0	2	0.25
Non-elective	34	41	0.40
Neurosurgery	26	22	0.66
Elective	23	19	0.63
Non-elective	3	3	1.00
Others	37	31	0.53
Elective	18	16	0.86
Non-elective	19	15	0.60

This table shows the distribution of subjects between the control and filter groups by demographic characteristics, PIM II and disease categories on admission. Admissions were subdivided into elective (previously planned admission) and non-elective admissions. None of the differences between the two groups were significant

Data are presented as the mean ± standard deviation (SD), or as the number (*n*) of patients, where indicated

^ a^ *P* values were calculated using the *t* test for equality of means, Pearson’s chi-Square test or Fisher’s exact test, as appropriate

For the reporting of adverse events, see ESM.

### Primary endpoints

**Fig. 2:**
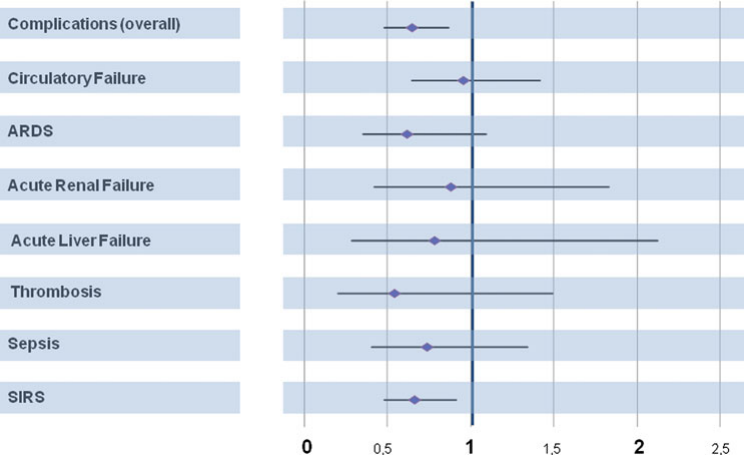
Hazard ratios of primary objectives for the treatment effect of in-line filtration. The incidence of overall complications and systemic inflammatory response syndrome (*SIRS*) were significantly reduced in the filter group. A trend towards a reduction in acute respiratory distress syndrome (*ARDS*) was evident for the filter group (*P* = 0.08). No significant differences were found for the incidence of sepsis, circulatory failure, acute renal failure, acute liver failure and thrombosis.* Filled rhombi* Hazard ratios, *horizontal lines* 95 % confidence intervals

**Fig. 3:**
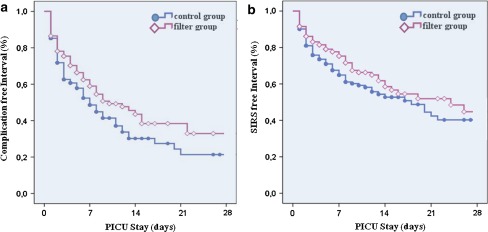
Kaplan–Meier analysis for complication-free interval (**a**) or SIRS-free interval (**b**). Control (*blue filled circle*) and filter (*red open rhombus*) group (maximum PICU stay 28 days). *Circles* and *rhombi* indicate censored patients

In-line filtration significantly decreased the overall complication rate from 40.9 % (*n* = 166) in the control group to 30.9 % (*n* = 124) in the filter group (*P* = 0.003), (Table 2; Fig. 2). The Kaplan–Meier method and log-rank test were used to analyze the time to first occurrence of any complication per patient (Fig. 3), and a significant difference between the control and filter group was found using the log-rank test (*P* = 0.003). The median of event-free time for the control group (7.0 ± 0.2 days) differed from that for the filter group (10.0 ± 1.9 days). The incidence of SIRS was significantly reduced in the filter group compared to that in the control group (22.4 [*n* = 90] vs. 30.3 % [*n* = 123], respectively; *P* = 0.01). Adjustment to the baseline risk calculation (PIM II) was performed for overall complication rate and SIRS without any relevant impact on statistical significance (Table 2). The Kaplan–Meier analysis revealed a significant difference in SIRS-free interval for the proportions of patients of both groups (*P* = 0.011 by the log-rank test) (Fig. 3). Although there were reductions in the incidence of sepsis, ARDS, circulatory failure, thrombosis, acute liver and acute renal failure in the filter group, these differences did not reach statistical significance (Table 2; Fig. 2).

**Table 2:** Morbidity outcomes

Characteristics	Control group (*n* = 406)	Filter group (*n* = 401)	*P* value^a^	95 % Confidence interval
Primary objectives (*n*)				
Complications (overall)	166	124	0.003	0.484−0.865
Adjusted to PIM II			0.011	0.502−0.914
SIRS	123	90	0.011	0.485−0.913
Adjusted to PIM II			0.026	0.500−0.958
Sepsis	27	20	0.313	0.406−1.337
Circulatory failure	60	57	0.593	0.604−1.334
ARDS	35	22	0.082	0.354−1.069
Acute renal failure	16	14	0.736	0.425−1.831
Acute liver failure	9	7	0.631	0.289−2.125
Thrombosis	11	6	0.230	0.200−1.489
Secondary objectives				
Mortality (*n*)	27	16	0.093	0.309−1.100
Length of stay (days)^b^	3.89 (2.96−4.81)	2.98 (2.33−3.63)	0.025	
Duration of mechanical ventilation (h)^b^	14.0 (5.6−22.4)	11.0 (7.1−14.9)	0.028	

Comparison of primary and secondary outcomes between control and filter group

*ARDS* Acute respiratory distress syndrome,* SIRS* systemic inflammatory response syndrome

^a^ *P* values were calculated using Pearson’s Chi-Square test, Fisher’s exact test or log-rank test as indicated

^b^Data are presented as the median with the range given in parenthesis

### Secondary endpoints

The length of stay of the filter group in the PICU was significantly reduced (23 %) compared to that of the control group (2.98 [95 % confidence interval 2.33−3.64] vs. 3.89 [2.97−4.82] days, respectively; *P* = 0.025) (Table 2). Although statistically not significant, overall hospital stay was reduced by 1 day in the filter group verus the control group (15.0 [13.4−16.6] vs. 16.0 [14.2−17.8] days, respectively; *P* = 0.19). The duration of mechanical ventilation was significantly reduced in the filter group versus that in the control group (11.0 [7.1−14.9] vs. 14.0 [5.6−22.4] h, respectively; *P* = 0.028). There was a statistical trend towards lower mortality in the filter group (6.7 [*n* = 27] vs. 4.0 % [*n* = 16], control vs. filter group; *P* = 0.09).

## Discussion

In this prospective clinical trial, in-line filtration of infusion solutions led to a significant decrease in major complications in critically ill pediatric patients. A significant reduction from 40.9 to 30.9 % in the overall complication rate of severe events (SIRS, sepsis, circulatory failure, ARDS, thrombosis, acute renal failure and acute liver failure) was demonstrated for the filter group. Although sample size and power were not calculated to detect reduction in single complications, the incidence of SIRS was significantly reduced. Additionally, all other primary objectives had a lower incidence in the filter group, but without statistical significance. In-line filtration significantly decreased both the duration of mechanical ventilation and the length of stay in the PICU. Considering the relatively low incidence of mortality in the PICU compared to an adult cohort, the statistical trend towards a reduction in mortality rate in the filter group is especially noteworthy.

This is the first clinical trial involving more than 800 PICU patients to reveal a significant benefit of in-line filtration on serious complications. However, our results seem to be in contrast to a recent Cochrane analysis including 704 neonates and preterm infants of four different trials, which demonstrated no favorable effect of in-line filtration on morbidity or mortality [26]. Two of the four studies included in the Cochrane analysis focused on thrombophlebitis and cannula patency, and only one study reported on endpoints comparable to those chosen for our study [27]. In this latter study, the authors demonstrated a significant reduction in the incidence of typical neonatal complications for in-line filtration, which is consistent with our data. In the fourth study of van Hoogen et al. [28], the primary objective was a reduction of sepsis, which, similar to our results, could not be significantly reduced by the use of in-line filters.

In our trial, the majority of severe complications occurred within the first 3 days following admission to the PICU, with 40.9 % of patients in the control group experiencing at least one major complication during this period. Implementation of in-line filters as early as the time of admission prevented severe complications and generated a persistent reduction in the complication rate (Fig. 3). These protective effects of in-line filtration may be explained by the preservation of microcirculation in all organs. The maintenance of microcirculation is particularly crucial in critically ill patients to prevent organ failure, whereas several frequently coexisting pathologies, such as inflammation and trauma, compromise the microcirculation [18]. The early restoration of microcirculation shown for inflammatory syndromes such as sepsis is associated with reduced morbidity and lower organ failure score [29]. In the pathological state of an already reduced microcirculatory perfusion, infused particles may cause additional impairment, leading to a loss of capillary density, as demonstrated in recent preclinical studies [14, 15]. Thus, the threshold of organ recovery is exceeded, and clinical signs of organ dysfunction or failure arise.

Based on our data, in-line filtration is effective in preventing SIRS in intensive care patients. SIRS was first used in adults to describe a nonspecific systemic inflammatory process in the absence of infection [21, 30]. SIRS criteria for adults were incorporated and modified with age-specific norms for children by the IPSCC [21]. SIRS in children is manifested by the presence of at least two of four criteria, one of which must be an abnormal temperature or leukocyte count: (1) hyperthermia or hypothermia, (2) tachycardia, (3) tachy- or bradypnoea or (4) leukocytosis or leukopenia [21]. The occurrence of SIRS predisposes patients to organ failure and frequently determines clinical outcomes [30]. Mortality and morbidity of severe non-infectious SIRS does not differ from those of severe sepsis, as shown in a multicentre trial involving 3,500 patients admitted to adult intensive care units [31]. The incidence of SIRS has been found to be higher than that of sepsis in both adult [31] and pediatric [32] intensive care patients. SIRS on admission has been shown to determine mortality and the length of stay of critically ill trauma patients [33]. In a prospective survey of 3,708 hospitalized adults, patients with SIRS had a 26 % chance of developing sepsis [34]. In another study, the more SIRS criteria fulfilled by a patient, the more likely that patient was to develop ARDS, disseminated intravascular coagulation or acute renal failure or even die of SIRS [33]. Pathophysiologically, mechanisms of SIRS and sepsis are similar, but the management of SIRS is typically non-specific [35]. Only supportive intensive care management, including fluid resuscitation, mechanical ventilation, inotropic support combined with the treatment of the initiating insult, might be beneficial [35]. In this setting of limited therapeutic options, in-line filtration represents a potent strategy to prevent SIRS and associated co-morbidities.

Although sample size and power were not calculated to detect a reduction in ARDS, a statistical trend towards a reduction was evident for the in-line filtration group. Consistent with this, a significantly decreased duration of mechanical ventilation was found, supporting the hypothesis that the lung as the physiological filter of intravenous infusion is most vulnerable to particulate damage. The mechanical obstruction of capillaries in the lung and other organs by infused particles has been demonstrated in both clinical and experimental studies [7, 12, 14]; such obstruction may impair coagulation through the consumption of pro- and anticoagulative proteins and platelets [7]. In vitro incubation with endothelial cells and macrophages demonstrated immunomodulating effects of particles [5]. Similar mechanisms have been shown for deposits of inhaled particles in the lung, which initiate a local inflammatory process that expands into a systemic inflammatory response with an increase in circulating inflammatory factors and activation of immune cells [36].

We adhered to a standardized infusion regimen, prevented incompatibilities and had access to CIVA and we still achieved a further reduction in the incidence of complications by using in-line filtration. The medical staff received extensive training in the handling of in-line filters prior to the initiation of our study, followed by continuous support by the authors during the study period. This practice enabled the implementation of filters without any relevant problems and demonstrated the practicability of the standard infusion arrangement and filter set-up. Thus, during the entire trial period there were only an irrelevant number of blocked filters and no serious adverse events of in-line filtration were noted. To ensure maximum patient safety in drug administration, filter membranes were visible in order to control for imminent blockage or defects. This compulsory open label study design may be a limitation and a potential source of bias.

Consistent with results reported in other trials [33], we have demonstrated a correlation between SIRS and length of stay in the PICU. Based on our data, one would expect that applying an in-line filter would also reduce diagnostic and therapeutic resources and increase turnover on the PICU. However, economic aspects of in-line filtration were not an endpoint in our study. As already shown by a meta-analysis [26], additional costs of in-line filters are compensated for by the reduced consumption of intravenous administration sets [27, 28] and decreased time for changing infusion sets [28]. In both of these studies [27, 28], intravenous sets in the control group were changed daily, while in the in-line filter group (interventional group) the changing times were extended up to 96 h. Due to different changing intervals in our study—72 h in both the control and filter group—these calculations are only partially applicable. Costs for disposables in our infusion set-up amounts to an additional expense of approximately 30 Euros for filters plus about 2 min extra work for equipment set-up. By lengthening the changing time from 72 to 96 h, some of the additional cost could be amortized. In summary, diagnostic and therapeutic resource-saving effects are likely to outweigh the additional costs for the filters and, consequently, positive economic effects can be expected.

In conclusion, the results of our trial demonstrate the safety and efficacy of in-line filtration in preventing major complications in patients admitted to the PICU. The overall complication rate and the incidence of SIRS were reduced among those patients with in-line filtration, indicating that filtration is a preventive strategy that can result in decreased morbidity of critically ill patients, reduced duration of mechanical ventilation and reduced length of stay on the PICU. Furthermore, in-line filtration was shown to improve the safety of infusion therapy. Further research is necessary to fully elucidate the pathophysiological mechanisms underlying our clinical findings.

## Electronic supplementary material

Below is the link to the electronic supplementary material.

Supplementary material 1 (DOC 90 kb)

## Abbreviations

ARDS: Acute respiratory distress syndrome
CIVA: Centralized intravenous additive service
PICU: Pediatric intensive care unit
PIM II: Pediatric Index of Mortality II
SIRS: Systemic inflammatory response syndrome
