# Supplementary material for: In-line filtration reduces severe complications and length of stay on pediatric intensive care unit: a prospective, randomized, controlled trial
Source: Intensive Care Med. 2012 Apr 12;38(6):1008–16. doi: 10.1007/s00134-012-2539-7 (PMC3351606; doi:10.1007/s00134-012-2539-7)
Supplement: Supplementary file 1 — Supplementary material 1 (DOC 90 kb) [file 134_2012_2539_MOESM1_ESM.doc]

**ESM - Electronic Supplementary Material**

This appendix has been provided by the authors to give readers additional information about their work.

**Figure 1:** Standard infusion setup and filter positioning


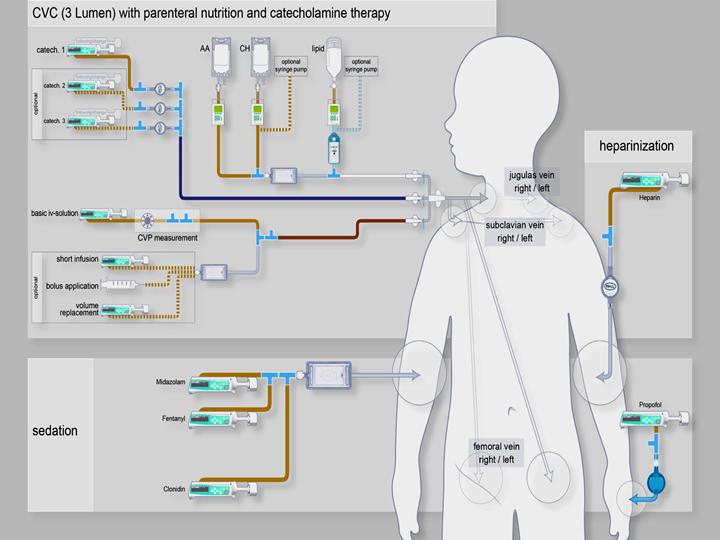


**Fig 1.** Standard for the application of infusions and medications and the positioning of in-line filters processed for the patients allocated to the filter group. The same standard for iv-access and the infusion set-up without in-line filters was used for the control group.

**MATERIAL AND METHODS**

**Definition of Endpoints**

Concisely, SIRS is defined as the presence of at least two of the following four criteria, one of which must be an abnormal temperature or leukocyte count (For further details please see electronic supplementary appendix): Core temperature of >38.5°C or <36°C; tachycardia defined as mean heart rate >2 SD above normal for age, mean respiratory rate >2 SD above normal for age, leukocyte count elevated or depressed for age or >10% immature neutrophils. Sepsis was defined as SIRS in the presence of or as a result of suspected or confirmed infection [20,21]. Thrombosis was defined as central venous catheter-related partial or complete blockage of a blood vessel lumen by a mural thrombus diagnosed by ultrasound or angiography. The criteria for acute liver failure were determined by the Paediatric Acute Liver Failure Study group [22]. Patients with a) no evidence of chronic liver disease, b) biochemical evidence of acute liver injury and c) coagulopathy defined as international normalized ratio (INR) ≥1.5 in the presence of hepatic encephalopathy or INR ≥2.0 regardless of encephalopathy met these criteria. ARDS was defined according to the American-European Consensus Conference as a condition with severe hypoxaemia (ratio of partial pressure of arterial oxygen (PaO_2_) to fraction of inspired oxygen (FiO_2_) <200) and bilateral infiltration seen on frontal chest radiograph in the absence of heart failure or primary pulmonary disease [23]. Circulatory failure was characterized by the need for any vasoactive or inotropic drug (epinephrine, norepinephrine, dobutamine, dopamine) to maintain blood pressure above the 5^th^ age-specific percentile [20]. The definition of acute renal failure was based on the Paediatric-modified Risk, Injury, Failure, Loss, End-stage kidney disease (RIFLE) criteria [24] which define acute renal failure by the estimated creatinine clearance (decrease by 75% or below 35ml/min/1.73m²) or by urine output (<0.5ml/kg/h for 24h or anuria for >12h).

Secondary endpoints were reduction of length of stay on the PICU and overall hospital stay. Patients were defined as dischargeable from the PICU when they were no longer in need for vital organ support according to standardized protocol. Finally, mortality rates and duration of mechanical ventilation were assessed for both groups. Extubation criteria were defined according to a strict standardized protocol to assess extubation readiness.

**Statistical Analysis**

Baseline characteristics of the two groups were compared using the t-test for equality of means. Disease categories, primary and secondary endpoints were compared using Pearson’s Chi-Square test or Fisher’s exact test when statistically indicated. All tests were two-sided. Results were considered to indicate statistical significance at a P value of less than 0.05. Data for length of stay (LOS) and duration of mechanical ventilation (MV) are reported as medians and range, all other data as means ± SD (95% CI). The Kaplan-Meier method and log-rank test were used to analyze the complication free and SIRS free interval for both groups. In the survival analyses, individuals who died or were discharged from the PICU were censored at the time of the event. The log-rank test was applied to compare duration of mechanical ventilation, length of stay on the PICU and overall hospital stay. There was no imputation of missing data. Statistical analysis was performed with the use of Predictive Analysis Software for Windows (SPSS / PASW), version 18.

**Data collection**

On admission, demographic and clinical data were obtained and recorded in different databases. Paediatric index of mortality (PIM) II [25] was calculated for each patient on admission. Blood chemical studies and haematologic tests were routinely performed at the time of admission, daily and when clinically required.

For further information see ESM.

Relevant clinical data were registered for each patient at least every hour. In case criteria for primary or secondary endpoints were met, this was recorded for each patient. The database was thoroughly checked for consistency. Any queries were resolved and the final database entries were verified by investigators blinded for the allocation of the patients. Serious adverse events were recorded according to standard definitions (<http://www.fda.gov/medwatch/report/DESK/advevnt.htm>).

**RESULTS**

**Adverse Events / Safety**

In one case at the beginning of the study, the administration of a high glucose concentration (70%) delivered at a low infusion rate blocked a filter membrane which led to transient hypoglycaemia in one infant without any further impairment. In this case, due to the technical specifications of the infusion pump system, no pressure alarm occurred when the intravenous line became clogged. The blockage was primarily noticed when nurses periodically checked the filters.
